# Redox Sensor Array with 23.5-μm Resolution for Real-Time Imaging of Hydrogen Peroxide and Glutamate Based on Charge-Transfer-Type Potentiometric Sensor

**DOI:** 10.3390/s21227682

**Published:** 2021-11-18

**Authors:** Tatsuya Iwata, Yuki Okumura, Koichi Okumura, Tomoko Horio, Hideo Doi, Kazuhiro Takahashi, Kazuaki Sawada

**Affiliations:** 1 Department of Electrical and Electronic Information Engineering, Toyohashi University of Technology, Toyohashi 4418580, Japan; Okumura-Y10@mail.dnp.co.jp (Y.O.); okumura-k@int.ee.tut.ac.jp (K.O.); horio-t@int.ee.tut.ac.jp (T.H.); doi.hideo.sy@tut.jp (H.D.); takahashi@ee.tut.ac.jp (K.T.); sawada@ee.tut.ac.jp (K.S.); 2 Department of Electrical and Electronic Engineering, Toyama Prefectural University, Imizu 9390398, Japan

**Keywords:** bioimaging, redox sensor array, potentiometry, H_2_O_2_, glutamate

## Abstract

Towards clarifying the spatio-temporal neurotransmitter distribution, potentiometric redox sensor arrays with 23.5-µm resolution were fabricated. The sensor array based on a charge-transfer-type potentiometric sensor comprises 128×128 pixels with gold electrodes deposited on the surface of pixels. The sensor output corresponding to the interfacial potential of the electrode changed logarithmically with the mixture ratio of K${}_{3}$Fe(CN)${}_{6}$ and K${}_{4}$Fe(CN)${}_{6}$, where the redox sensitivity reached 49.9 mV/dec. By employing hydrogen peroxidase as an enzyme and ferrocene as an electron mediator, the sensing characteristics for hydrogen peroxide (H${}_{2}$O${}_{2}$) were investigated. The analyses of the sensing characteristics revealed that the sensitivity was about 44.7 mV/dec., comparable to the redox sensitivity, while the limit of detection (LOD) was achieved to be 1 µM. Furthermore, the oxidation state of the electron mediator can be the key to further lowering the LOD. Then, by immobilizing oxidizing enzyme for H${}_{2}$O${}_{2}$ and glutamate oxidase, glutamate (Glu) measurements were conducted. As a result, similar sensitivity and LOD to those of H${}_{2}$O${}_{2}$ were obtained. Finally, the real-time distribution of 1 µM Glu was visualized, demonstrating the feasibility of our device as a high-resolution bioimaging technique.

## 1. Introduction

Neurotransmitters (NTs) such as acetylcholine (ACh), dopamine (DO), glutamate (Glu), and γ−amino butyric acid (GABA) are chemical messengers for signal transmission between synapses in central nerve systems (CNS), playing an important role in brain function including behavior and cognition [1]. Their irregular concentrations that affect the synaptic transmission are considered to be linked to various diseases such as Alzheimer’s, Parkinson’s disease, schizophrenia, and depression [2]. The concentration of NTs dynamically changes due to their release from a synapse followed by diffusion, reuptake, and enzyme degradation [3,4]. Therefore, clarifying the relationship between the extracellular spatio-temporal distribution of NTs and human behavior is of great importance for diagnosis, leading to the development of remedies for these diseases [5,6].

For these purposes, various methods for the detection of NTs were developed [3,7,8,9]. Among them, electrochemical (EC) sensors were extensively studied due to their potential to fast response, label-free, high-sensitivity, low-cost, and easy-to-use testing systems [10]. In previous reports, EC detection of NTs listed above: ACh [11,12,13,14,15,16], DO [17,18,19,20,21,22], Glu [15,23,24,25,26,27,28,29,30,31], and GABA [32,33,34,35], was reported. These EC devices were improving their limit of detection (LOD) by modifying their electrode materials and structures, and demonstrated submicromolar LOD [14,21,22]. More recently, electrochemical sensor arrays attracted attention to capturing the spatio-temporal concentration of NTs. EC sensors were advancing their resolution and pixel density by utilizing microfabrication technology to achieve sub-mm order spatial resolution [36,37,38,39,40,41,42]. Tedjo et al. fabricated 4096-channel microelectrode array (MEA) with the spatial resolution of 25.5μm×30.4μm, and reported the detection of catecholamines down to 8 μM. Dudina et al. integrated 9216 carbon nanotube field-effect transistors into an array. They demonstrated Glu detection down to the concentration 10 μM by a single channel. Although these reports achieved the pixel number of the order of 1000 with the pixel pitch of the order of 10 μm, the amperometric sensor arrays generally suffer from the reduction in signal current with decreasing the area of working electrodes. Since the reduction in the signal current is directly linked to the deterioration of LOD, that fact imposes difficulty in achieving a higher spatial resolution to the level required to capture the NT dynamics, which is considered to be the order of 1 μm [4], while keeping low LOD.

On the contrary, potentiometric sensors detect the interfacial potential of a working electrode, which follows the Nernst equation. Their output signal ideally does not change with the element size, and thus, they are advantageous for miniaturized sensor arrays. In particular, we were developing electrochemical imaging techniques based on charge-transfer-type (CTT) potentiometric sensor arrays. The sensors fabricated based on CMOS technology comprises 128×128 pixels with spatial and temporal resolution of 23.5 μm and 33 ms, respectively, and demonstrated real-time pH imaging [43]. The array enabled the real-time imaging of pH changes in a brain tissue in vivo [44]. In a recent development, the sensor array with 256×256 pixels shrunk its pixel area down to 2 μm with the temporal resolution of 0.5 ms [45], showing the potential as a high spatio-temporal pH imaging technique. It was also demonstrated that the array applies to the detection of biomolecules including ACh [46] and ATP [47,48], based on acid generation by an enzymatic reaction. Although the CTT sensors showed the potential for the imaging of NTs, they suffer from low output signals in the application to living organisms including cells and tissues [48]. This comes from the fact that the sensor utilized pH change to capture the signal of NTs. Body fluid generally shows buffer action, which suppresses the pH change, and hence, the output signal of the CTT sensors is significantly reduced. Against this problem, we adopted redox electrodes [49,50] and developed Glu sensors that are insensitive to pH change, combining the enzymatic reaction with the redox species [51,52]

In this study, toward further improvement in imaging quality and LOD of the array, NT sensing characteristics based on redox potential were investigated in detail, setting Glu, one of the major NTs, as a target material. The measurement results, including the sensitivity among the pixels and calibration curve, were analyzed in detail and the decisive factor for LOD was discussed. Additionally, the imaging results for Glu are presented.

## 2. Experimental Procedure

### 2.1. Redox Reaction and Sensor Output

In this study, we employed horseradish peroxidase (HRP) and glutamate oxidase (GluOx) as enzymes to degrade H${}_{2}$O${}_{2}$ and Glu, respectively. The enzyme-catalyzed reactions are described as follows [25]

(1) $$\begin{array}{cc} \mathrm{Glu}+O_{2}+H_{2}O & \overset{\mathrm{GluOx}}{\to }2-\mathrm{oxoglutarate}+{\mathrm{NH}}_{3}+H_{2}O_{2}, \end{array}$$

(2) $$\begin{array}{cc} H_{2}O_{2}+2\mathrm{Fc}+2H^{+} & \overset{\mathrm{HRP}}{\to }2H_{2}O+2{\mathrm{Fc}}^{+}. \end{array}$$

In Equation (2), Fc and Fc${}^{+}$ indicate ferrocene in reduced and oxidized states, respectively. The potential of the Au layer $E_{\mathrm{Au}}$ is determined by the ratio of the electron mediators, and for diluted solution, well described as [49,53]

(3) $$E_{\mathrm{Au}}=E_{\mathrm{Au}}^{o}+\frac{RT}{F}\ln\frac{\left[{\mathrm{Fc}}^{+}\right]}{\left[\mathrm{Fc}\right]},$$

where $E_{\mathrm{Au}}^{o}$ is the potential in the standard state, *R* the gas constant, *T* temperature, and *F* Faraday constant. Square brackets in the second term indicate the concentration of the species. The output of each pixel in the sensor array ($V_{\mathrm{Out}}$) is related to $E_{\mathrm{Au}}$ as

(4) $$V_{\mathrm{Out}}=E_{\mathrm{Au}}+C,$$

where *C* is related to the interfacial potential of the reference electrode and others and can be regarded as a constant during the measurements. Hence, the changes in $V_{\mathrm{Out}}$ in response to the addition of substances (H${}_{2}$O${}_{2}$ or Glu in this study) correspond to the changes in $E_{\mathrm{Au}}$ as

(5) $$\Delta V_{\mathrm{Out}}=\Delta E_{\mathrm{Au}}.$$

### 2.2. Device Fabrication

**Figure 1:**
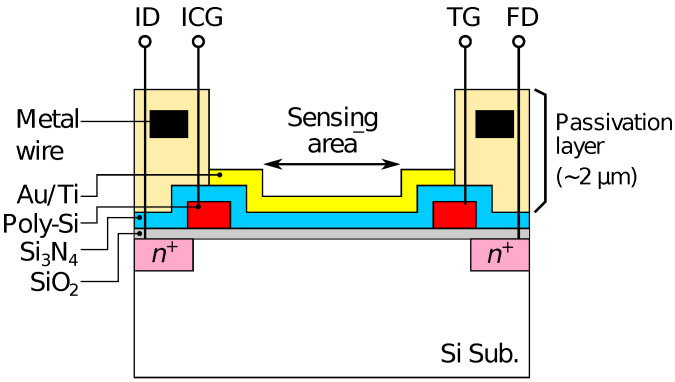
Schematic illustration of pixel structure of fabricated device.

The redox sensor array was fabricated based on a CTT potentiometric sensor array. The potentiometric sensor array is fabricated based on complementary metal-oxide-semiconductor (CMOS) technology, and has 128 × 128 pixels with a pitch of 23.5 μm. The detailed structure and operational principle of the potentiometric sensor array are described elsewhere [43]. As the schematic cross-section of the pixel structure shown in Figure 1, a 20-nm-thick gold (Au) film with a 5-nm-thick titanium adhesion layer was evaporated on the sensing area of the array to form a redox electrode. For the detection of H${}_{2}$O${}_{2}$ and Glu, Fc was used as an electron-mediator. The enzymes were immobilized by a poly-ion-complex (PIC) membrane, where poly-L-lysine (PLL) and poly(sodium 4-styrenesulfonate) (PSS) were employed as a polycation and a polyanion, respectively [51]. The PIC membrane was deposited by a conventional layer-by-layer method [54]. Firstly, a 10 μL of 60 mM PLL solution was dropped and dried for 10 min at room temperature (RT). Then, an enzyme solution containing 10 units of HRP and GluOx was dropped and dried at 4 ${}^{\circ }$C overnight. Finally, 10 μL of 75 mM PSS was dropped and dried for 1 h at RT.

### 2.3. Materials

A recording medium (RM) composed of 135 mM NaCl, 5 mM KCl, 2 mM CaCl${}_{2}$, 1 mM MgCl${}_{2}$, 10 mM D-glucose, and 10 mM sodium 4-(2-hydroxyethyl)piperazine-1-ethanesulfonate (HEPES), where these substances were dissolved in deionized water (DIW) (18 MΩcm at 298 K), was used to prepare sample solutions that mimic biological environments. To prepare the RM containing Fc, ferrocenyl methanol (FcMeOH) was employed. FeMeOH was first dissolved in ethanol, and then, the solution was mixed into the RM. On the other hand, the mixture of potassium hexacyanoferrate(III) [K${}_{4}$Fe(CN)${}_{6}$] and potassium hexacyanoferrate(II) [K${}_{3}$Fe(CN)${}_{6}$] was also used to examine the redox response of the sensors.

HRP, GluOx, and FcMeOH (95%) were purchased from Sigma–Aldrich Inc. Sodium HEPES (≥99%) was purchased from Dojindo Laboratries. NaCl, KCl, MgCl${}_{2}$, H${}_{2}$O${}_{2}$ (30.0%), ethanol (99%), K${}_{4}$Fe(CN)${}_{6}$·3H${}_{2}$O (99.5%), and K${}_{3}$Fe(CN)${}_{6}$ (99.0%) were purchased from Wako Pure Chemical Industries, Ltd.

### 2.4. Measurement Procedure

**Figure 2:**
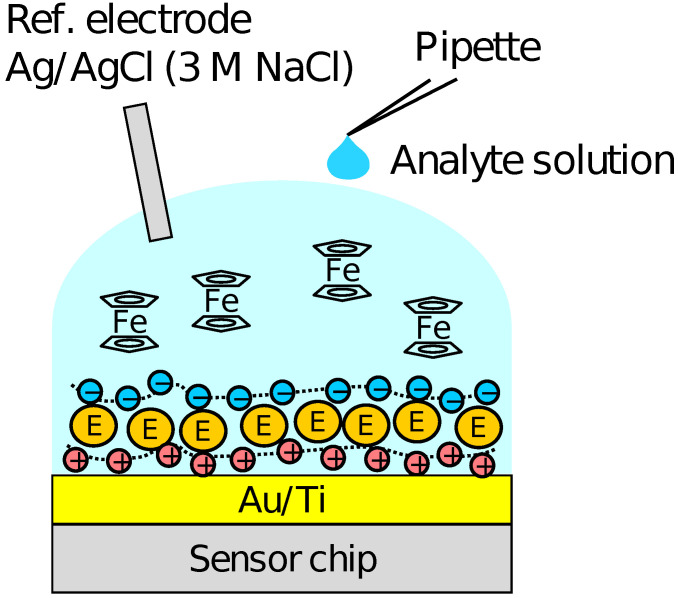
Schematic illustration of setup for H${}_{2}$O${}_{2}$ and Glu measurements.

Firstly, the output of the sensor without the PIC membrane was measured using the solution of K${}_{3}$Fe(CN)${}_{6}$ and K${}_{4}$Fe(CN)${}_{6}$ with various mixture ratios to examine the redox response. Then, the response to H${}_{2}$O${}_{2}$ was measured in the sensor with the enzymes immobilized and Fc as a mediator. The schematic illustration of the measurement setup is shown in Figure 2. A 90 μL of the RM containing 500 μM FcMeOH was first put on the array, and then, 10 μL of sample solution containing H${}_{2}$O${}_{2}$ and 500 μM FcMeOH was added dropwise. The measurements were carried out for the H${}_{2}$O${}_{2}$ concentration range of 10${}^{-8}$–10${}^{-4}$ M. After the measurements for each of the concentrations, the sample solution was removed from the surface each time and the sensor surface was washed by the RM several times. The output distribution among the pixels and the concentration dependence of the output were analyzed employing the output values at 300 s after the addition of H${}_{2}$O${}_{2}$, at which we assumed the enzymatic reactions were sufficiently progressed. The sensing characteristics of Glu were also measured with a similar procedure to that for H${}_{2}$O${}_{2}$ sensing. A reference electrode of Ag/AgCl with 3 M NaCl was used. Although KCl inner solution for Ag/AgCl reference electrode is advantageous in terms of ion mobility, the leakage of potassium ions from a high-density solution may be harmful taking into account the application to cell measurements. Therefore, 3 M NaCl was employed as inner solution in this study.

## 3. Results and Discussion

### 3.1. Fabricated Device

**Figure 3:**
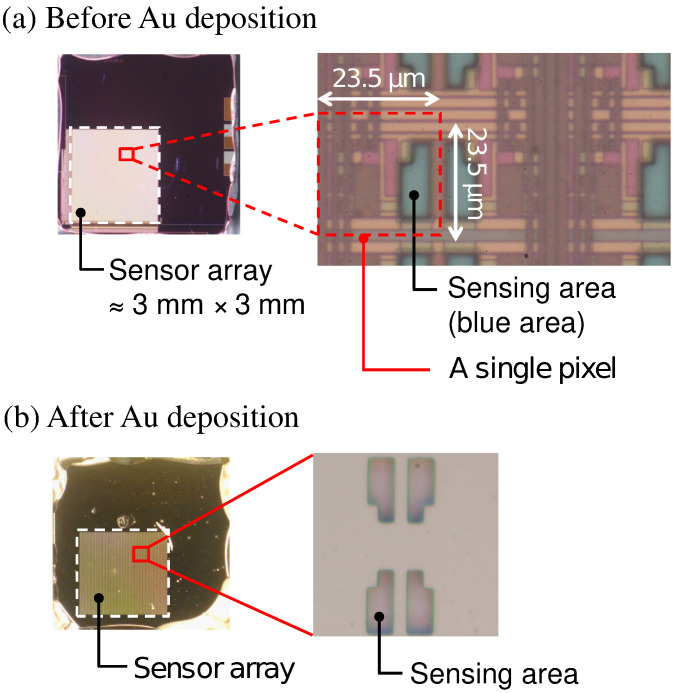
Picture of sensor chip (**a**) before and (**b**) after Au deposition.

Optical microscopy images of the sensor chip (a) before and (b) after Au deposition are shown in Figure 3. The bluish region shown in Figure 3a corresponds to the sensing area. In Figure 3b, it was observed that the Au layer was deposited in each of the sensing areas. As schematically depicted in Figure 1, the surface of the sensing area is lowered than the surrounding areas due to the passivation layer. The flux of Au vapor incident into the sensor surface was nearly perpendicular to the sensing area, and thus, the thickness of an Au layer deposited on the sidewalls was negligible. As a result, the Au layer deposited on the pixels was separated from that on the neighboring pixels. The incident of the vapor can be regarded as nearly perpendicular to the sensor surface in terms of the deposition on the sidewall but not completely perpendicular, which caused the so-called shadowing effect, as will be discussed later.

### 3.2. Redox Sensitivity of the Sensor

**Figure 4:**
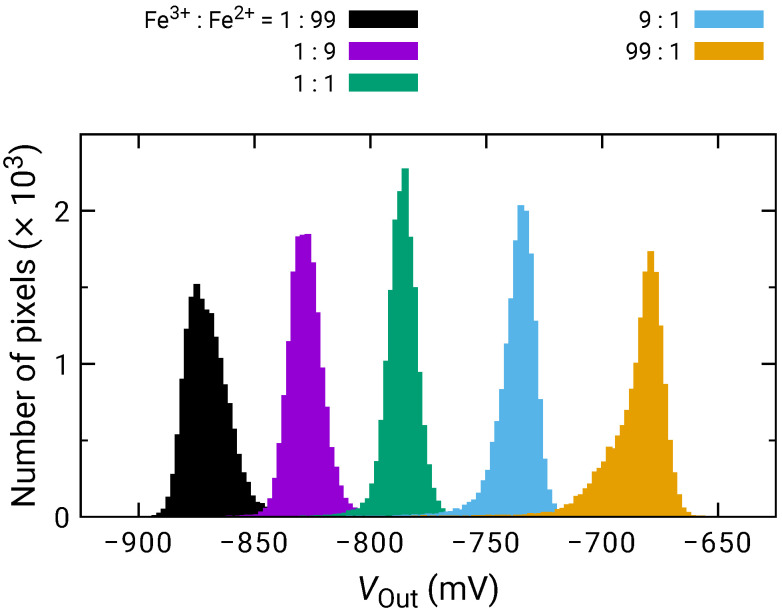
Histogram of $V_{\mathrm{Out}}$ for various ratio between K${}_{3}$Fe(CN)${}_{6}$ (Fe${}^{3+}$) and K${}_{4}$Fe(CN)${}_{6}$ (Fe${}^{2+}$).

**Figure 5:**
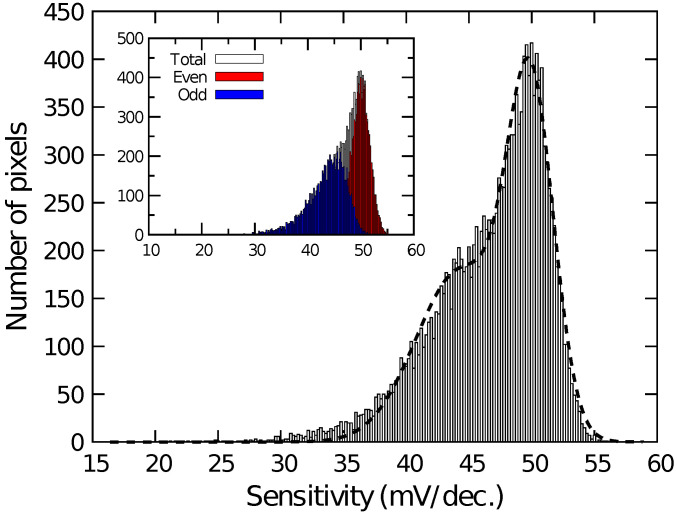
Histogram of redox sensitivity among pixels. Inset depicts sensitivity of even and odd columns.

Figure 4 shows the histogram of $V_{\mathrm{Out}}$ among the pixels for the different quotient of K${}_{3}$Fe(CN)${}_{6}$ and K${}_{4}$Fe(CN)${}_{6}$ (Fe${}^{3+}$/Fe${}^{2+}$). $V_{\mathrm{Out}}$ among pixels was calibrated for Fe${}^{3+}$/Fe${}^{2+}$ = 1:1. As the ratio of K${}_{3}$Fe(CN)${}_{6}$ increased, $V_{\mathrm{Out}}$ increased proportionally to the logarithm of Fe${}^{3+}$/Fe${}^{2+}$. Then, the sensitivity to the redox species was extracted on each pixel and its histogram is plotted in Figure 5. The histogram exhibited a peak near 50 mV/dec., while a shoulder peak near 45 mV/dec. was also observed. As the sensitivity was plotted for odd and even columns in the inset of Figure 5, the difference in the sensitivity between the even and odd column was observed, indicating the higher and lower peaks originate from the sensitivity of the even and odd columns, respectively. In general, the variation due to random processes should follow Gaussian distribution. Hence, assuming that the distributions corresponding to the even and odd columns have the same pixel numbers, the total distribution was fitted by the sum of two Gaussian distributions as:

(6) $$F\left(x\right)=\frac{N\Delta x}{2}\left\{\frac{1}{\sigma_{1}\sqrt{2\pi }}\exp\left(-\frac{{\left(x-m_{1}\right)}^{2}}{2\sigma_{1}^{2}}\right)+\frac{1}{\sigma_{2}\sqrt{2\pi }}\exp\left(-\frac{{\left(x-m_{2}\right)}^{2}}{2\sigma_{2}^{2}}\right)\right\},$$

where $m_{1}$ and $m_{2}$ are the average values and $\sigma_{1}$ and $\sigma_{2}$ are the standard deviation corresponding to each distribution. *N* is the total number of pixels and Δx is the class interval of the histogram (here, 0.2 mV/dec.). The function well fitted into the experimental result, as shown as a dashed curve in Figure 5. The extracted average values and standard deviations for each of the Gaussian distributions are derived as $m_{1}=49.9$ mV/dec., $\sigma_{1}=1.9$ mV/dec., $m_{2}=44.4$ mV/dec., and $\sigma_{2}=3.6$ mV/dec., revealing that the redox sensitivities were slightly smaller than the Nernst limit (59.1 mV/dec at 298 K).

**Figure 6:**
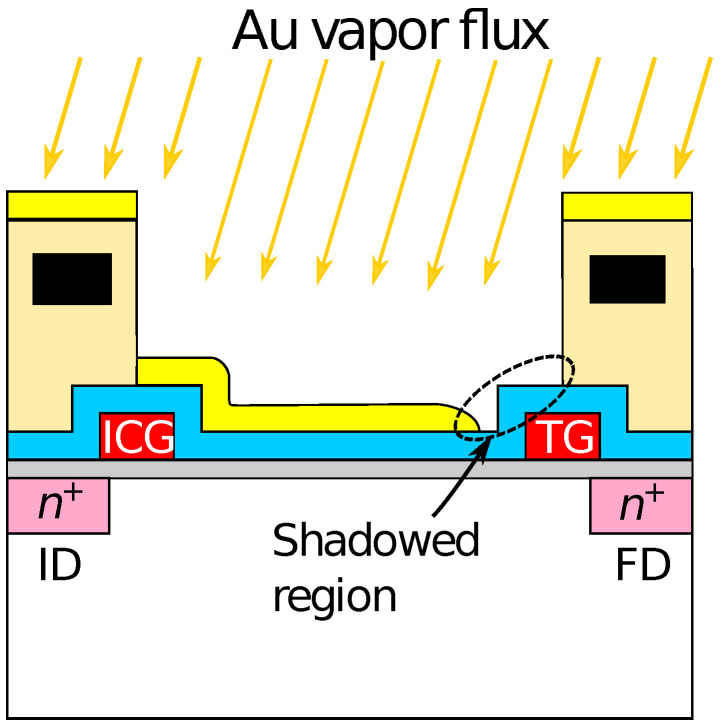
Schematic illustration of shadowing effect during evaporation of gold electrode.

Then, the smaller redox sensitivity together with the sensitivity difference between the even and odd columns is discussed. In the device, the interfacial potential of the Au electrode according to the mixture ratio of redox species determines the depth of the potential well in the semiconductor part under the sensing area (see Figure 1). Charges are stored in the potential well, and then transferred to a floating diffusion amplifier (FD) through a transfer gate (TG), whereby the charges corresponding to the redox potential are converted to $V_{\mathrm{Out}}$ [43]. Therefore, the potential well corresponding to the areas that are not covered with the Au layer is insensitive to the redox potential, reducing the stored charge. More critically, the coverage near TG affects the transferring efficiency of the charges. If the area near the TG is not covered with the Au layer, the potential well nearby TG is only modulated by the fringing field due to the TG potential, causing the degradation of the transferring efficiency of the charges to FD, thus, $V_{\mathrm{Out}}$. The insufficient coverage may be caused by a shadowing effect during the evaporation taking into account the pixel structure, because the surface of the sensing area is lower by approximately 2 μm than the surrounding area as schematically shown in Figure 6. As a result, the insufficient Au layer coverage, which degraded the conversion efficiency of the redox potential to $V_{\mathrm{Out}}$, resulted in the lower redox sensitivity compared with that of the Nernst limit.

Additionally, the sensor pixel in the even and odd columns have a symmetric layout, namely, the relative location of TG, which caused the different shadowing effect during the evaporation, resulting in the different redox sensitivity. Although it was suggested that the shadowing effect during the film deposition can cause sensitivity variation among the pixels considerably, this problem will be solved by a flatter pixel structure that was recently developed [45]. In the structure, it was employed the so-called extended gate structure [53]. The roughness over the entire sensing area is less than 100 nm, for which the shadowing effect should be negligible, taking account of the pixel pitch of 2 μm. NT sensing based on the new-flatter structure is now under investigation.

### 3.3. H${}_{2}$O${}_{2}$ Sensing Characteristics

**Figure 7:**
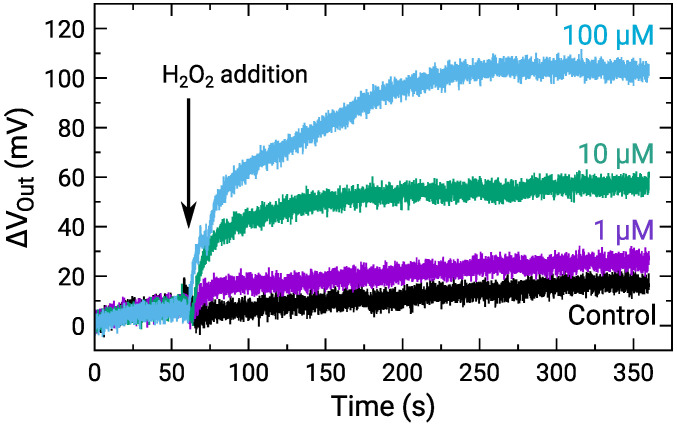
Time-dependent output change for various H${}_{2}$O${}_{2}$ concentrations, where control indicates addition of RM without H${}_{2}$O${}_{2}$. Sample solutions containing H${}_{2}$O${}_{2}$ were added at approximately 60 s as indicated by an arrow.

The sensor response to H${}_{2}$O${}_{2}$ for the concentration range $10^{-8}–10^{-4}$ M was measured. Figure 7 shows the time-dependent $\Delta V_{\mathrm{Out}}$ of a center pixel of the array for various H${}_{2}$O${}_{2}$ concentrations, where $\Delta V_{\mathrm{Out}}$ was defined as the $V_{\mathrm{Out}}$ change from the onset of the measurements. The result for the control, for which RM without H${}_{2}$O${}_{2}$ was added, is also shown. The $\Delta V_{\mathrm{Out}}$ of all the H${}_{2}$O${}_{2}$ concentrations gradually increased similarly after staring the measurements due to the output drift. In contrast, after the addition of H${}_{2}$O${}_{2}$ at approximately 60 s, $\Delta V_{\mathrm{Out}}$ for 1 μM H${}_{2}$O${}_{2}$ and more significantly became larger than that for the control. Note that the temporal drop of $V_{\mathrm{Out}}$ at the H${}_{2}$O${}_{2}$ addition is an artifact [47]. Although its origin should be investigated, the difference in $\Delta V_{\mathrm{Out}}$ was observed among the H${}_{2}$O${}_{2}$ concentration. Then, we focus on the $\Delta V_{\mathrm{Out}}$ difference. The response times to reach 95 % of the saturation values were roughly estimated to be 10 s, 95 s, and 125 s for 1 μM, 10 μM, and 100 μM, respectively. Although the response times are still long at this stage, it was suggested that it is mainly limited by the diffusion of the molecules inside into the enzyme-immobilizing membrane as reported recently [48]. The molecules instantly reach the sensor surface with a sufficiently thin enzyme membrane, and thus, the response time would be reduced. The improvements in the response time should be investigated to obtain the spatio-temporal distribution of NTs in the future.

**Figure 8:**
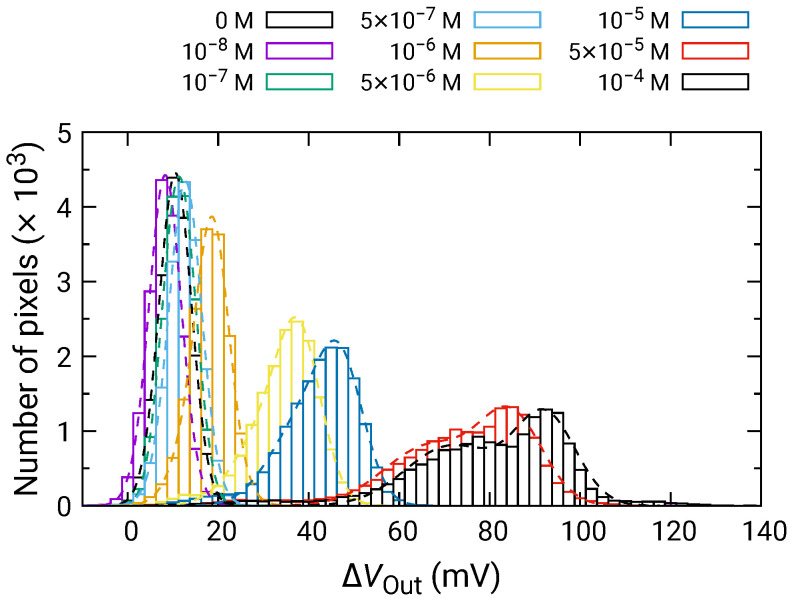
Histogram of $\Delta V_{\mathrm{Out}}$ in response to H${}_{2}$O${}_{2}$ addition.

Figure 8 shows the distribution of $\Delta V_{\mathrm{Out}}$ for each of the H${}_{2}$O${}_{2}$ concentrations depending on the H${}_{2}$O${}_{2}$ concentration. For 500 nM and below, $\Delta V_{\mathrm{Out}}$ was comparable to that for 0 M; hence, significant $V_{\mathrm{Out}}$ change was not observed. In contrast, the distribution of $\Delta V_{\mathrm{Out}}$ began to shift higher values for 1 μM and above, and $\Delta V_{\mathrm{Out}}$ became larger as the H${}_{2}$O${}_{2}$ concentration increased. Here, $\Delta V_{\mathrm{Out}}$ larger than 0 V for 0 M H${}_{2}$O${}_{2}$ is due to the output drift, as observed in Figure 7.

**Figure 9:**
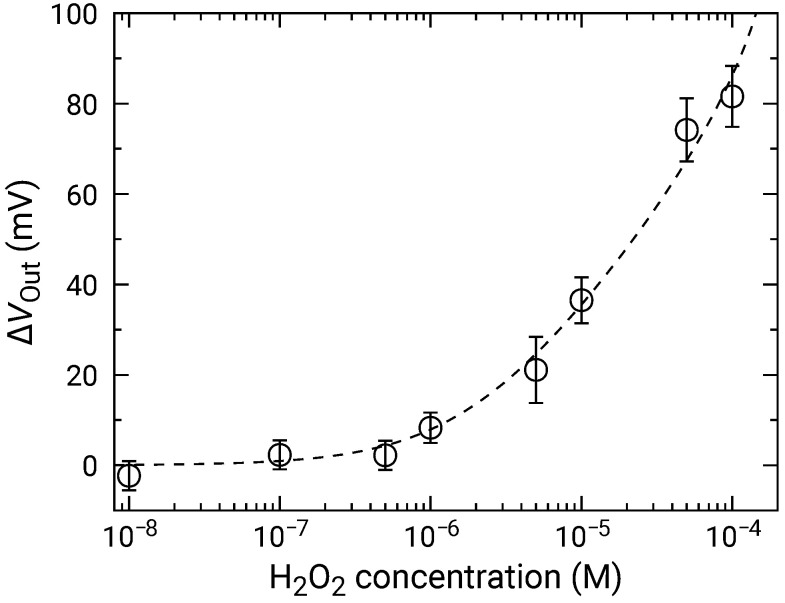
Concentration dependence of sensor output change for addition of H${}_{2}$O${}_{2}$. Data are plotted after subtracting $\Delta V_{\mathrm{Out}}$ for 0 M H${}_{2}$O${}_{2}$ (control) to compensate for output drift.

At high concentrations, the distribution became broader, and two distinct peaks were observed (e.g., 50 and 100 μM). As discussed in Section 3.2, the broad distribution with a shoulder peak is attributed to the variation in the redox sensitivity among the pixels. Then, using Equation (6), each of the distributions was fitted, and the fitting result is shown as dashed curves. The average values and standard deviations of the distributions were extracted from the fitting. As discussed in Section 3.2, the histogram exhibited two peaks, which originated from the variation in the redox sensitivities among the pixel. In this study, the average values for the higher distribution were adopted for the following analyses to evaluate the sensitivity to H${}_{2}$O${}_{2}$. The average values of $\Delta V_{\mathrm{Out}}$ are shown in Figure 9 as a function of H${}_{2}$O${}_{2}$ concentration, where $\Delta V_{\mathrm{Out}}$ for 0 M was subtracted from $\Delta V_{\mathrm{Out}}$ for each of the concentrations to omit the influence of the output drift. The error bars in the figure represent the standard deviation extracted from the fitting of the histogram. As described above, significant $\Delta V_{\mathrm{Out}}$ was observed at 1 μM and above, showing that the LDO was in the order of 1 μM for both samples.

Then, the concentration dependence of $\Delta V_{\mathrm{Out}}$ is analyzed in detail. $V_{\mathrm{Out}}$ is determined by the quotient of the concentration of oxidized and reduced species as described in Equation (3). Then, $\Delta V_{\mathrm{Out}}$ during the measurements is described as [50]:

(7) $$\begin{array}{cc} \Delta V_{\mathrm{Out}} & =V_{\mathrm{Out}}-V_{\mathrm{Out}},0 \end{array}$$

(8) $$\begin{array}{cc} \; & =\frac{RT}{F}\ln\frac{\left[{\mathrm{Fc}}^{+}\right]\left[{\mathrm{Fc}}_{0}\right]}{\left[\mathrm{Fc}\right]\left[{\mathrm{Fc}}_{0}^{+}\right]}, \end{array}$$

where the subscript 0 indicates their initial values (i.e., before H${}_{2}$O${}_{2}$ addition). Assuming that the reaction involving H${}_{2}$O${}_{2}$ sufficiently proceeded, the concentration of ferrocene is related to H${}_{2}$O${}_{2}$ concentration, and then, setting $V_{s}$ as the effective redox sensitivity, $\Delta V_{\mathrm{Out}}$ becomes

(9) $$\Delta V_{\mathrm{Out}}=V_{s}\log\frac{\left(\left[{\mathrm{Fc}}_{0}^{+}\right]+2\left[H_{2}O_{2}\right]\right)\left[{\mathrm{Fc}}_{0}\right]}{\left(\left[{\mathrm{Fc}}_{0}\right]-2\left[H_{2}O_{2}\right]\right)\left[{\mathrm{Fc}}_{0}^{+}\right]}.$$

Here, the theoretical limit of $V_{s}$ is 59.1 mV at 298 K. Assuming $\left[{\mathrm{Fc}}_{0}^{+}\right]\ll \left[{\mathrm{Fc}}_{0}\right]$, the total concentration of ferrocene $\left[{\mathrm{Fc}}_{\mathrm{Tot}}\right]$ is approximated as $\left[{\mathrm{Fc}}_{\mathrm{Tot}}\right]=\left[{\mathrm{Fc}}_{0}\right]+\left[{\mathrm{Fc}}_{0}^{+}\right]\approx \left[{\mathrm{Fc}}_{0}\right]$. Then, Equation (9) is

(10) $$\Delta V_{\mathrm{Out}}=V_{s}\log\frac{\left(\left[{\mathrm{Fc}}_{0}^{+}\right]+2\left[H_{2}O_{2}\right]\right)\left[{\mathrm{Fc}}_{\mathrm{Tot}}\right]}{\left(\left[{\mathrm{Fc}}_{\mathrm{Tot}}\right]-2\left[H_{2}O_{2}\right]\right)\left[{\mathrm{Fc}}_{0}^{+}\right]}.$$

Setting $V_{s}$ and $\left[{\mathrm{Fc}}_{0}^{+}\right]$ as fitting parameters, the experimental results were fitted by Equation (10), where $\left[H_{2}O_{2}\right]$ was a variable. The results were shown as dashed curves in Figure 9. The extracted parameters are $V_{s}=44.7\pm 4.4\;\mathrm{mV}/\mathrm{dec}.$ and $[F_{c_{0}}^{+}]=4.0\pm 1.3\;\mu M$, respectively. The errors of extracted parameters are not those originating from the distribution, but are fitting errors for the data shown in Figure 9.

Although the extracted $V_{s}$ is slightly smaller than the redox response of 49.9 mV/dec, they are comparable to each other taking account of the range of error, which indicates that that the H${}_{2}$O${}_{2}$ was fully degraded and that the ferrocene acted as the electron mediator. On the other hand, $\left[{\mathrm{Fc}}_{0}^{+}\right]$ was the order of ∼$10^{-6}$ M in all the samples. Given that the LOD was the same order as $\left[{\mathrm{Fc}}_{0}^{+}\right]$, it may determine the LOD of the samples in this study. The redox potential of ferrocene is approximately +0.64 V vs. NHE [55], and thus, $\left[{\mathrm{Fc}}^{+}\right]$ is negligible under equilibrium in the RM we employed (pH was approximately 7.1). However, it was reported that Ferrocene can be oxidized or reduced under light irradiation [50]. A similar situation might occur in this study, although further investigation is necessary. As a consequence, $\left[{\mathrm{Fc}}^{+}\right]$ should be reduced in the initial state of the measurements to improve LOD. This, conversely, indicates that the LOD might be further improved by the optimal choice of redox species and by appropriate treatment before the measurements. Ishige et al. [50] improved the LOD of FET sensors with ferrocenyl-alkanethiol modified electrode by fully oxidizing the ferrocene before detecting the reducing species. Similarly, the reduction procedure before the measurements might be effective against our devices.

### 3.4. Application to Glutamate Imaging

**Figure 10:**
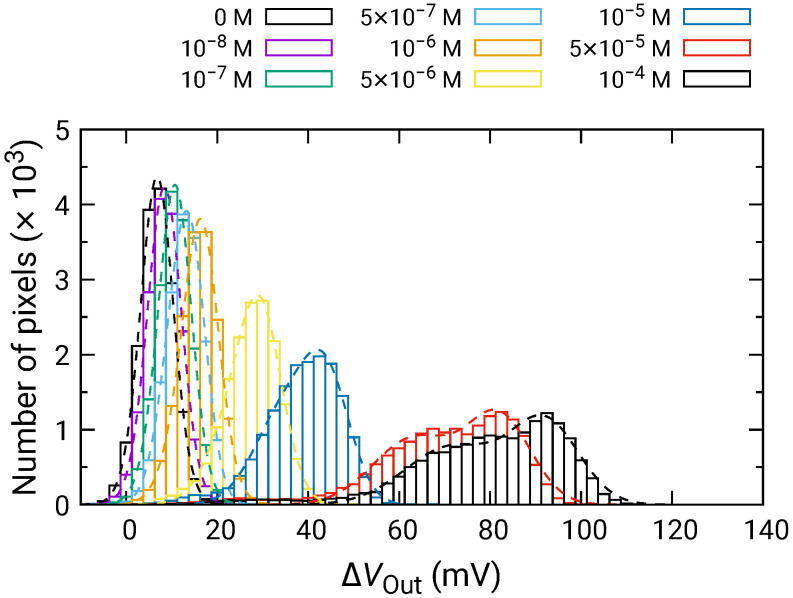
Histogram of $\Delta V_{\mathrm{Out}}$ in response to Glu addition.

**Figure 11:**
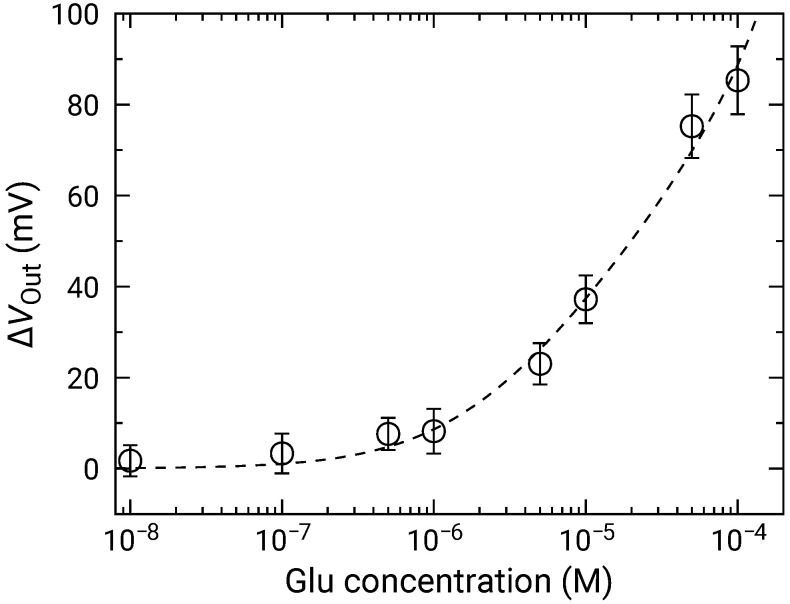
Concentration dependence of sensor output change for Glu addition. Data are plotted after subtracting $\Delta V_{\mathrm{Out}}$ for 0 M Glu (control) to compensate for output drift.

Figure 10 shows the $\Delta V_{\mathrm{Out}}$ distributions for various Glu concentrations. In a similar manner to those for H${}_{2}$O${}_{2}$, the distribution exhibited two distinct peaks. Then, the fitting was carried out as shown by dashed curves, and extracted parameters for the higher peaks are plotted as a function of concentration in Figure 11. The concentration dependence analyzed by Equation (10) where $\left[\mathrm{Glu}\right]$ was used as the variable instead of $\left[H_{2}O_{2}\right]$. $V_{s}$ was approximately 45.0±3.6 mV/dec., which was almost similar to that for H${}_{2}$O${}_{2}$. This result indicates that the Glu was fully degraded into H${}_{2}$O${}_{2}$, and that the influences of the products including 2-oxoglutarate and ammonia on the redox reactions are negligible. Additionally, the LOD of 1 μM was also achieved.

**Figure 12:**
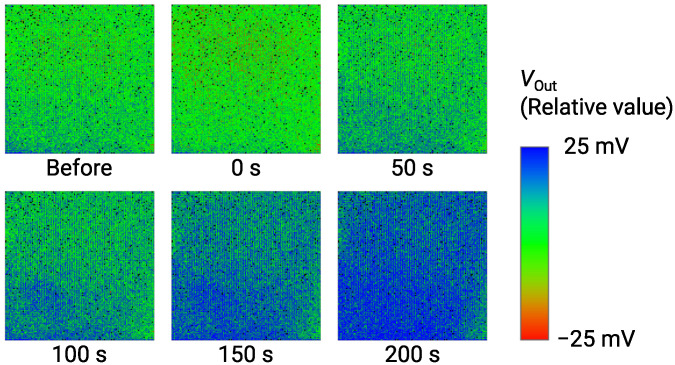
Time-dependent change of output image for addition of 1 μM Glu. Labels below images indicate elapsed times after addition of Glu.

The imaging result of $\Delta V_{\mathrm{Out}}$ for 1 μM Glu is obtained as shown in Figure 12. $V_{\mathrm{Out}}$ is shown as a relative value over a range of 50 mV by a color bar. Just after the Glu addition, $V_{\mathrm{Out}}$ was decreased by approximately 5 mV, which was an artifact due to the specimen addition process as described in Section 3.3 (See Figure 7). Then, the color image gradually changed from green to blue due to the increase in $V_{\mathrm{Out}}$ as time evolves. At 200 s, the image became bluish over the almost entire area, indicating the significant output change was obtained as an image. As a result, we successfully demonstrated the imaging of Glu down to 1 μM. Table 1 compares the performance of the sensor array with previous studies [26,42,56]. This work simultaneously achieves high-spatial-resolution and good LOD. On the required LOD, it is reported that the extracellular Glu concentration reaches mM range, while the baseline is much lower down to 25 nM [57]. Therefore, the sensors with the LOD of 1μM may apply to capturing the Glu release [58]. Nevertheless, taking into account such a low baseline, further lower LOD is still necessary, for which the approaches for improving the LOD discussed in Section 3.3 should be investigated as future work.

Finally, the repeatability of the sensor and the influence of interfering substances are discussed. As described in Section 2.4, the measurements were carried out for each of the H${}_{2}$O${}_{2}$ and Glu concentrations by changing the sample solution each time. Between each measurement, the sensor surface was washed by RM several times. According to the procedure, the sensor experienced 16 successive measurements (8 measurements for H${}_{2}$O${}_{2}$ and Glu). As a result, similar concentration dependence was obtained between these substances as shown in Figure 9 and Figure 11, indicating the repeatability of the sensor response and that the activity of the enzyme was not lost. Although the interference from other substances is not examined at this stage, redox species (e.g., ascorbic acid) other than target molecules should affect the response taking into account the mechanism of the sensor, which should be an inevitable issue. The sensor in this study comprises a lot of sensor elements with a small pitch (23.5 μm in the current sensor) and is beneficial to implementing multi-analyte sensing by immobilizing the different enzymes among neighboring pixels, like pixels in a color camera [59]. Similarly, the influence of the interfering molecules could be addressed by fabricating the sensor pixels on which the enzyme was immobilized or not. Thereby, the responses by purely the interfering substances and those superimposed with the response of the target molecules are obtained at different pixels. By analyzing these responses from the neighboring pixels, at which the concentration of the molecules can be regarded as the same, the responses from the target molecules can be deconvoluted, and therefore, the responses of interfering substances could be excluded.

## 4. Conclusions

In this study, redox electrodes were implemented on a CTT potentiometric sensor array with 128×128 pixels as a pH-insensitive method for NTs sensing. The redox electrodes comprised gold electrodes and ferrocene as an electron mediator. The redox sensitivity characterized using the mixture of K${}_{3}$Fe(CN)${}_{6}$ and K${}_{4}$Fe(CN)${}_{6}$ was confirmed to reach 49.9 mV/dec., while it was found to be affected by the coverage of the gold electrode on the sensing area. Then, H${}_{2}$O${}_{2}$ sensing characteristics were investigated. From the calibration curve for the output voltage, the LOD was estimated to be around 1 μM. The analyses of the calibration curve revealed that the sensitivity was 44.7 mV/dec., being comparable with that confirmed by the mixture of K${}_{3}$Fe(CN)${}_{6}$ and K${}_{4}$Fe(CN)${}_{6}$. Additionally, it was suggested that the control of the oxidation state of the redox mediator is the key to further improving LOD. Finally, as a result of the Glu sensing measurements, the LOD of 1 μM and sensitivity, comparable to the H${}_{2}$O${}_{2}$, was obtained. Furthermore, the real-time imaging of 1 μM Glu was demonstrated, showing the promising property of the device fabricated in this study as a promising bioimaging device for clarifying the spatio-temporal distribution of NTs in CNS.

## Figures and Tables

**Table 1:** Comparison of performance of sensor arrays for Glu sensing.

Method	Pixel Pitch	Number of Elements	LOD	Ref.
Amperometry	550 μm (probe pitch)	4 (probe array)	0.5 μM	[56]
Amperometry	200 μm	2	0.6 μM	[26]
Amperometry	22.5/15 μm	9216	10 μM	[42]
Potentiometry	23.5 μm	16384	1 μM	This work
